# Diagnosing Cardiac Amyloidosis: From Heart Failure to Electrical Storm

**DOI:** 10.1155/2021/3293728

**Published:** 2021-06-19

**Authors:** Adithya T. Mathews, Abu-Sayeef Mirza, Chandrashekar Bohra, Akshay G. Mathews, Philip Ritucci-Chinni, Jamie L. Weber, William W. Bulkeley, Maqsood Siddique, David Whitaker, Richard Bowerman

**Affiliations:** ^1^Department of Internal Medicine, University of South Florida, Tampa, FL, USA; ^2^University of South Florida, Tampa, FL, USA; ^3^Department of Pathology, James A Haley Veteran Affairs Hospital, Tampa, FL, USA; ^4^Department of Cardiology, James A Haley Veteran Affairs Hospital, Tampa, FL, USA

## Abstract

Cardiac amyloidosis is a condition when amyloid fibers are deposited in the extracellular space of the heart causing tachyarrhythmias, heart failure, or sudden cardiac death. We present a 71-year-old woman presenting with dyspnea on admission. Echocardiogram revealed diastolic heart failure and left ventricular hypertrophy with strain pattern concerning for an infiltrative process. She was discharged with diuretic therapy and scheduled for a cardiac magnetic resonance imaging. One week after discharge, she was readmitted with progressive shortness of breath and syncope. She was found to be in shock and had multiple episodes of cardiac arrest with both ventricular tachycardia and pulseless electrical activity. She developed electrical storm and eventually passed within 24 hours. Autopsy revealed gross cardiomegaly and left ventricular hypertrophy with Congo red staining revealing amyloid fibrils with apple-green birefringence. This case demonstrates the rapid progression of cardiac amyloidosis from acute-onset diastolic heart failure to uncontrollable ventricular tachycardia, and eventually death. We review the literature regarding multiple diagnostic modalities that facilitate the confirmation of cardiac amyloidosis.

## 1. Introduction

Amyloidosis is the extracellular tissue deposition of amyloid fibrils consisting of low molecular weight subunit proteins [1, 2]. Cardiac amyloidosis is considered an infiltrative cardiomyopathy associated with an unfavorable prognosis [2]. The two major types of cardiac amyloidosis include acquired monoclonal immunoglobin light chain (AL) and transthyretin (ATTR). ATTR also has two different forms between acquired type (ATTRm) and inherited type (ATTRwt). The pathophysiology involves normal protein transforming into an amyloid state, due to cleavage, denaturation, or excess production [3]. Eventually, these proteins form in an antiparallel beta-pleated sheet configuration, becoming amyloid fibrils [2, 4]. Two-thirds of AL cardiac amyloidosis patients meet Mayo stage III disease on diagnosis with the basis of elevated N-terminal probrain natriuretic peptide (NT-proBNP) and troponin-I. These elevated markers are associated with death within the first few months of diagnosis [5]. However, clinical outcome often depends on the type of amyloid fibril deposits and how much tissue is involved [2]. We discuss a difficult case highlighting the importance of diagnostic modalities for cardiac amyloidosis that quickly progressed from acute heart failure to electrical storm.

## 2. Case Presentation

A 71-year-old Caucasian woman with a history of hypertension, hyperlipidemia, type 2 diabetes mellitus, stage 1A right invasive ductal carcinoma pT1cPN0M0, and melanoma (diagnosed in 2000 status after left midback excision) had presented to the emergency department with 2 weeks of worsening dyspnea on exertion. On exam, she had jugular venous distension (JVD) and bilateral leg edema with labs remarkable for brain natriuretic peptide (BNP) of 1714 pg/mL (normal <100 pg/mL), D-dimer 2295 ng/mL (normal <500 ng/mL), and elevated troponin of 0.21 ng/mL (normal <0.04 ng/mL).

**Figure 1:**
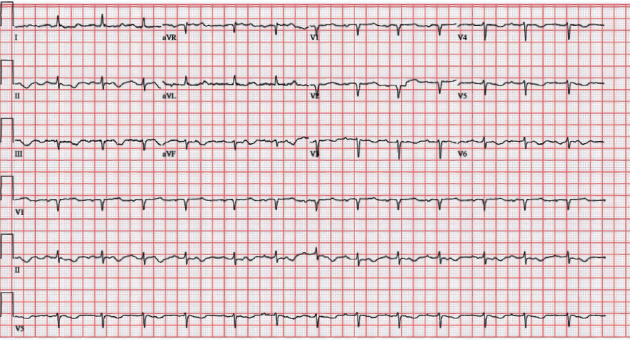
Electrocardiogram on admission showing sinus rhythm with low voltage, prior infarct of anteroseptal regions, and T-wave inversions in lateral leads.

**Figure 2:**
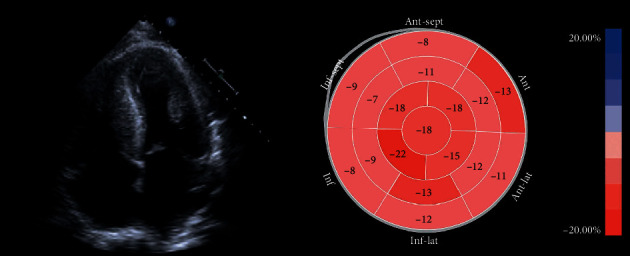
Echocardiography shows low global longitudinal cardiac strain (−13.4%) with “cherry on top” pattern (normal value for global longitudinal strain is −20% and below in healthy individuals). Strain pattern is suggestive of possible cardiac amyloidosis.

Electrocardiogram revealed sinus rhythm with first-degree AV block, low voltage, old infarct with Q waves in anteroseptal leads, and T-wave inversions in anterolateral leads (Figure 1). Echocardiogram revealed grade III restrictive diastolic filling pattern suggestive of increased left atrial pressure, preserved ejection fracture (60–65%), dilated inferior vena cava with <50% respiratory variation, and severe concentric left ventricular hypertrophy concerning for infiltrative process per strain pattern (Figure 2). Interventricular septum was 1.5 cm (normal: 0.7–1.1 cm), and posterior wall thickness was 1.4 cm (normal: 0.7–1.1 cm). The wall motion was normal with no suggestion for prior focal myocardial infarction. She also had a serum calcium >11 mg/dL (normal <8.6 mg/dL), with an elevated globulin gap (4.3 g/dL on admission), and serum protein electrophoresis revealed an IgG lambda monoclonal peak (2683 mg/dL) (normal: <1600 mg/mL) with an abnormal kappa/lambda light chain ratio with kappa of 5.37 mg/dL (normal: <1.96 mg/dL) and lambda of 6535 mg/dL (normal: <1.96 mg/dL) concerning for plasma cell dyscrasia.

She later received a bone marrow biopsy which revealed 60% cellular marrow with 40% plasma cells consistent with plasma cell myeloma, but further staining was not performed. A skeletal survey also revealed lytic lesions in bilateral humeri and femurs. She was treated with diuretics on her initial admission, and the patient sought early discharge from the hospital. She was discharged with a diuretic for management of heart failure with preserved ejection fraction with recommendations for a cardiac magnetic resonance imaging and close follow-up with cardiology and oncology.

**Figure 3:**
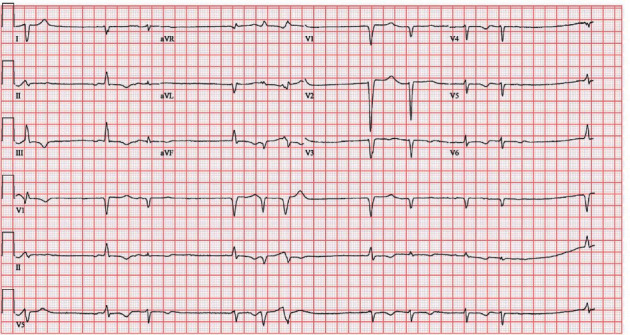
Electrocardiogram after cardiac arrest with junctional bradycardia and premature ventricular complexes.

**Figure 4:**
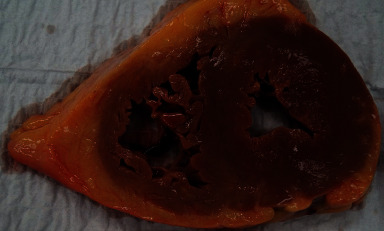
Autopsy of cardiac muscle with increased subpericardial fat asymmetrically concentrated more towards the right ventricle compared to the left ventricle.

**Figure 5:**
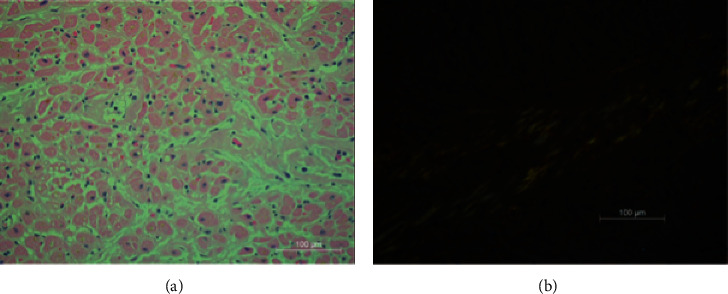
(a) Infiltrative amyloid deposits noted among the myocytes on hematoxylin and eosin stain. (b) Congo red stain with apple-green birefringence within the right and left ventricle tissues.

One week after discharge, she was readmitted with progressive shortness of breath, fevers, syncope, as well as a clinical presentation consistent with early cholecystitis. Laboratory studies revealed WBC >21 × 10^9^/dL (normal: <9.9 × 10^9^/dL), acute renal failure with a creatinine level of 1.9 mg/dL (normal: <1.1 × mg/dL), shock liver with aspartate aminotransferase 495 U/L (normal: <40 U/L) and alanine aminotransferase 494 U/L (normal: <40 U/L), BNP of 4687 pg/mL (normal: <100 pg/mL), troponin of 2.82 ng/mL (normal: <0.04 ng/mL), and potassium of 4.6 mEq/L (normal: 3.5–5.2 mEq/L). Abdominal computed tomography scan revealed new abdominal ascites and small gallbladder stones with pericholecystic stranding and duodenal thickening. On the day after admission, she developed unstable ventricular tachycardia but spontaneously converted after two minutes to junctional bradycardia (Figure 3). Telemetry strips were not saved from the event. Given concern for marked sinus bradycardia and corrected QT interval of 509 ms (normal <440 ms), R-on-T phenomena was suspected as a possible cause for the patient's polymorphic ventricular tachycardia. Intravenous isoproterenol was started, and advanced cardiac life support protocol was initiated. After 2 minutes of cardiopulmonary resuscitation (CPR), patient awoke but was unable to maintain saturation, and she was promptly intubated. Subsequent defibrillation converted the ventricular tachycardia to a sinus rhythm with a rate of 50–60 beats per minute. There was a concern for recurrent polymorphic ventricular tachycardia. Immediate left heart catherization revealed nonobstructive coronary artery disease. Right heart catherization showed an elevated mean right atrial pressure of 30 mmHg, right ventricular pressure of 62/17 mmHg, mean pulmonary artery pressure of 51 mmHg, pulmonary capillary wedge pressure of 40 mmHg, cardiac index of 1.8 L/min/m^2^, cardiac output of 3.2 L/min, and systemic vascular resistance of 920 dynes/seconds/cm^5^. A transvenous pacer wire was placed to assist against further ventricular tachycardia episodes. She appeared to have a mixed picture of distributive and cardiogenic shock. She remained hypotensive and in shock despite maximized dosing for norepinephrine, dopamine, and vasopressin. The patient decompensated again with unstable monomorphic ventricular tachycardia. Rhythm control agents with the use of amiodarone and overdrive pacing were futile. The ventricular tachycardia persisted despite several rounds of CPR, defibrillation, epinephrine, amiodarone, and bicarbonate. The patient's family came to the bedside and requested resuscitation efforts be terminated. They authorized the autopsy which subsequently revealed gross cardiomegaly and left ventricular hypertrophy (Figure 4). Congo red staining of both ventricular and hepatic tissue revealed amyloid fibrils with apple-green birefringence (Figure 5). Outside pathology report later confirmed AL amyloid deposition by liquid chromatography mass spectrometry.

## 3. Discussion

The patient had an unusual presentation of symptoms with rapid progression of heart failure and tachyarrhythmia. Her two hospitalizations were one week apart. While she was scheduled for outpatient workup for her plasma cell dyscrasia, this was unable to be completed due to her rapid progression and subsequent readmission. She developed cardiomyopathy, cardiogenic shock, and ventricular tachyarrhythmias that were nonresponsive to conventional therapies. Early recognition and diagnosis and treatment are vital for patients with AL cardiac amyloidosis.

Among the different types of amyloidosis, primary AL and both secondary amyloidosis ATTRm and ATTRwt can commonly involve the heart [2, 6]. Manifestations of cardiac amyloidosis include heart failure and heart block [7, 8]. Infiltration of amyloid fibrils in the ventricles results in stiffening progressing to restrictive cardiomyopathy causing diastolic dysfunction [2, 7, 8]. Cytotoxic effects of amyloid fibrils lead to apoptosis and fibrosis with eventual systolic dysfunction [2, 9]. The classic symptoms of heart failure manifest with exertional dyspnea, orthopnea, and lower extremity edema [2]. Atrial fibrillation is the most common arrhythmia with 10–20% incidence in all cases of cardiac amyloidosis [10]. Other common [11] conduction abnormalities include ventricular tachycardia and ventricular fibrillation [12]. Physical examination findings include those of heart failure including JVD, lower extremity edema, and an S3 gallop [2]. Extracardiac manifestations include purpura, macroglossia, periorbital edema, nephrotic syndrome, hepatomegaly, ascites, and bilateral carpal syndrome [2, 13].

There are various cardiac diagnostic modalities that facilitate the diagnosis of cardiac amyloidosis. A brief review of literature is presented as follows.

Electrocardiogram: cardiac amyloidosis manifests as pseudoinfarction pattern with low voltage in limb leads (<5 mm) and poor R-wave progression and precordial leads [11]. In AL, 50% have low voltage in frontal and precordial leads, whereas in ATTR, only 30% meet low voltage criteria (low voltage may be confined to frontal leads only) [14]. Cyrille et al. explains that the sum of amplitudes of S wave in V1 and R wave in V5 or V6 more than 3.5 mV is considered a measure of left ventricular hypertrophy [11] and less than 1.5 mV is associated with dismal outcomes in all cardiac amyloidosis [2, 15]. Absence of left ventricular hypertrophy does not exclude cardiac amyloidosis as only 10–20% will meet left ventricular hypertrophy criteria, while up to 50% will have pseudoinfarction pattern [14–16].

Echocardiogram: echocardiographic findings include biatrial enlargement, ventricular wall and valvular thickening, diastolic dysfunction, and classic granular sparkling appearance [2]. Doppler measurements include the assessment of ratio (E/A) of early (E) and late (A) diastolic peak velocities along with deceleration time (time taken by peak E velocity to return to baseline) [2]. Assessment of strain and strain rate (longitudinal axis dysfunction) can help diagnose cardiac amyloidosis earlier in subclinical stages and gauge survival outcomes [17]. The development of speckle-tracking echocardiography better analyzes the longitudinal axis, particularly radial and circumferential strain [2]. The significant decrease of longitudinal strain in the mid and basal wall regions with preservation of the apical region has high sensitivity and specificity (ranging from 90%–95% and 80%–85%, respectively) for cardiac amyloidosis [18]. Diffuse valve thickening, atrial septal thickening, and right ventricular hypertrophy help differentiate cardiac amyloidosis from other causes of left ventricular hypertrophy [19]. ATTR is common in aortic stenosis and can be seen in up to 15% of elderly patients with low-flow, low-gradient aortic stenosis [20]. The “cherry on top” strain pattern was found in our patient on the prior admission (Figure 2), but further workup was warranted for the diagnosis of cardiac amyloidosis.

Cardiac magnetic resonance imaging: cardiac magnetic resonance imaging employs various techniques including strain analysis and tissue imaging with and without contrast [2]. Cardiac magnetic resonance imaging can assess strain with a technique known as displacement encoding with stimulated echoes with high sensitivity and specificity rivaling echocardiography [21]. Myocardial injury secondary to amyloid deposition in the interstitium serves as a reservoir for gadolinium accumulation resulting in characteristic late gadolinium enhancement [22]. This technique has a sensitivity of close to 80% and specificity of 94% [23]. Late gadolinium enhancement when coupled with phase-sensitive inversion recovery has shown to provide a better assessment of the extent of cardiac involvement allowing Fontana [24] to classify patients with AL and ATTR in 3 subcategories (normal, subendocardium, and transmural) [2]. Transmural is associated with the worst prognosis (5.4-fold increase in mortality) and the progression of normal to subendocardium to transmural late gadolinium was proportionally related to extracellular volume expansion [24]. Although cardiac magnetic resonance imaging has extreme diagnostic utility, our patient decompensated acutely prior to obtaining this study.

Nuclear imaging: a noninvasive way to diagnose cardiac amyloidosis involves the accumulation of radiotracers, most commonly technetium-99m and 3,3-diphosphono-1,2-propanodicarboxylic acid (^99m^Tc-DPD) and technetium-99m-pyrophosphate (^99m^Tc-PYP) [2, 25]. ^99m^Tc-DPD scintigraphy has been useful in the workup for cardiac amyloidosis with high specificity and sensitivity for ATTR cardiac amyloidosis while showing low seismicity and high specificity for AL cardiac amyloidosis [26]. In addition to ^99m^Tc-DPD and ^99m^Tc-PYP, technetium-99m methylene diphosphonate (^99m^Tc-MDP) scintigraphy has also been studied in cardiac amyloidosis workup. All three forms of nuclear testing have shown such high sensitivity and specificity, especially when resulting with high uptake (Grade 2 or Grade 3) that additional endomyocardial biopsy would not be necessary for the diagnosis of ATTR cardiac amyloidosis [27]. Despite these imaging modalities, endomyocardial biopsy remains the gold standard given the ability to use microscopy and histopathology and directly observes the deposition of amyloid fibrils as amorphous deposits (as seen in our patient, Figure 5). Endomyocardial biopsy is a relatively safe procedure with almost 100% sensitivity [28]. However, our latent pathological diagnosis was from tissue obtained by autopsy.

The treatment modalities of cardiac amyloidosis are mainly aimed at managing clinical heart failure but specifically managing amyloidosis may involve chemotherapy and various transplant modalities including hematopoietic stem cell, heart, and liver transplant [7, 8, 29]. The role of heart transplant is still under investigation. Younger patients <60 years without associated plasma cell dyscrasia or other major organ involvement are better candidates for orthotopic heart transplant [2, 8].

## 4. Conclusion

Cardiac amyloidosis is a life-threatening manifestation of systemic amyloidosis. Patients with exertional dyspnea, low voltage electrocardiogram, and echocardiogram with left ventricular hypertrophy and diastolic dysfunction are concerning for cardiac amyloidosis. Cardiac magnetic resonance imaging with late gadolinium enhancement and phase-sensitive inversion recovery was initially ordered as the next step in our workup of cardiac amyloidosis. While supportive therapy for heart failure, chemotherapy and transplant are the currently available modalities for treatment, and the prognosis of cardiac amyloidosis depends upon the extent of myocardial involvement with amyloid. In addition, previously undiagnosed, advance-stage multiple myeloma was likely the precursor of our patient's cardiac amyloidosis.

Unfortunately, our patient progressed rapidly from heart failure to ventricular storm, thus underlining the importance of expediting advanced imaging diagnostics that may have facilitated expedient management.
